# Sexual and Life Satisfaction of Pregnant Women

**DOI:** 10.3390/ijerph17165894

**Published:** 2020-08-13

**Authors:** Dorota Branecka-Woźniak, Anna Wójcik, Joanna Błażejewska-Jaśkowiak, Rafał Kurzawa

**Affiliations:** 1 Department of Gynecology and Reproductive Health, Pomeranian Medical University of Szczecin, 71-210 Szczecin, Poland; ippblazejewska@gmail.com (J.B.-J.); kurzawa@pum.edu.pl (R.K.); 2 Clinic of Obstetrics and Gynecology, Independent Public Clinical Hospital No. 1, Pomeranian Medical University of Szczecin, 71-252 Szczecin, Poland; annawwojcik@onet.pl

**Keywords:** pregnancy, sexual satisfaction, life satisfaction

## Abstract

The awareness of one’s own sexuality and its expression depend on the stage of an individual’s life. Pregnancy is a period of many, also psychosexual, changes. The sexual needs of pregnant women are rarely discussed, and intercourse during this period seems to be a taboo. The aim of this study was to assess the sexual and life satisfaction of pregnant women. The study involved 181 pregnant women and was conducted from July to November 2018. The participants were patients of the pregnancy pathology ward, Independent Public Clinical Hospital No. 2 in Szczecin, and participants of antenatal classes. The research project was approved by the Bioethics Committee of the Pomeranian Medical University, Szczecin (KB-0012/74/18). This survey-based study was performed using the self-developed questionnaire and standardized tools: the Sexual Satisfaction Questionnaire, and the Satisfaction with Life Scale (SWLS). Statistical analysis was performed using the SPSS Statistics 24.0 statistical package. The tests used were the Kolmogorov–Smirnov test, the Shapiro–Wilk test, and Pearson’s test. The statistical significance of the results was assumed as (*p* < 0.05), the correlations (*p* < 0.01), and probability (*p* < 0.001). This study demonstrated a high level of life satisfaction, a medium level of overall sexual satisfaction, and a high level of satisfaction with sex. A higher level of satisfaction with life was associated with higher levels of sexual satisfaction in every dimension. There is a need for comprehensive perinatal care and professional sexual counseling.

## 1. Introduction 

The modern concept of health, based on the WHO definition, covers the physical, mental, social, spiritual, sexual, and reproductive aspects. Thanks to these values, it is possible to fulfill your aspirations and environmental activity. Sexual health and its relationship with other health components are an integral part of health understood in a holistic way. It means freedom from all forms of sexual violence, and is associated with the right to sexual identity and privacy, which allows individuals to engage in accordance with their needs, and thus display assertive sexual behavior. Sexuality is a natural function of the body, and one of the main factors motivating people to make contacts and bonds. At the same time, it is the outcome of many factors: biological, psychological, socio-cultural, ethical, religious, spiritual, and even political and legal. Therefore, it is impossible to find one definition that perfectly describes human sexuality. The perception of female sexuality has repeatedly changed over the centuries and depended on the cultural circle in which it was considered [1]. This is a complex issue determined by specific personal characteristics such as age, experience, and self-esteem. Contemporary social changes in the form of secularization and liberalization have an effect on the place of values related to sexuality, among them love, success, and the meaning of life [2]. The awareness of one’s own sexuality and the way of its expression are shaped over time, and the order of priority changes, depending on one’s period of life. 

The time of waiting for a child’s birth is a period of change in every sphere of a woman’s life, including physical and mental aspects, such as appearance, well-being, psyche, emotions, social roles, and sexuality. This favors the creation of myths about sexual activity in pregnancy, causing the anxiety of pregnant women and their negative attitudes. The sexual needs of pregnant women are rarely discussed, and intercourse during this period seems to remain a taboo. It is worth emphasizing, however, that in physiological, uncomplicated pregnancy, undertaking sexual activity is not only possible and natural, but also very beneficial to the sexual and mental health of both partners. A satisfying sex life maintains and/or improves the relationship and positively affects the self-esteem and well-being of a pregnant woman. It should, however, result from a conscious and unforced choice, and not the desire to meet the needs of the partner [3]. 

### Aim of the Study

We aimed to assess the sexual and life satisfaction of pregnant women.

## 2. Material and Methods

This study was conducted in a group of 181 pregnant women in different trimesters of pregnancy (1st, 2nd, and 3rd trimesters) from July to November 2018. The study included patients of the pregnancy pathology ward, Independent Public Clinical Hospital No. 2 in Szczecin, and participants of antenatal classes (Fryc’s Birth School, Katarzyna Zamiela’s Birth School, Birth School at the Family Medicine Center at Chopin street). The research project was approved by the Bioethics Committee of the Pomeranian Medical University, Szczecin (KB-0012/74/18).

This survey-based study was performed using the self-developed questionnaire and two standardized research instruments:

The self-developed questionnaire included 17 (both closed-ended and open-ended) questions divided into three parts: 1. sociodemographic data: age, education, marital status, place of residence, employment status; 2. pregnancy interview: the trimester of pregnancy, which pregnancy, having children, the age of children, the type of the previous delivery, the presence of a partner/husband during labor, doing Kegel’s exercises; 3. information about the relationship: partner’s age, the number of years lived together, the knowledge of intercourse, sources of this knowledge, the frequency of intercourse (decrease/increase in the frequency and its reasons).

The Sexual Satisfaction Questionnaire S1 (see the Supplementary Materials), developed by M. Plopa, is a standardized research instrument including ten statements about the intimate aspects of the relationship, divided into three dimensions: caress—two statements (satisfaction with touching each other, foreplay), closeness—six statements (openness, confiding, level of romantic relationship, acceptance of the fragrance), and sex—two statements (satisfaction with sexual intercourse, achieving orgasm). Respondents rate their satisfaction with the aforementioned aspects of sexual life on a five-point scale (A—no satisfaction, B—low satisfaction, C—adequate satisfaction, D—high satisfaction, E—maximum satisfaction). It is possible to indicate that a given activity is not currently in a relationship. The final score is a sum of points obtained for the three dimensions (caress, closeness, and sex). Raw results are converted into sten scores and interpreted as follows: 1–4—low level, 5–6—average level, and 7–10—high level of sexual satisfaction.

The Satisfaction with Life Scale (SWLS), developed by E. Diener, consists of five statements that respondents may agree or disagree with, rating answers on a seven-point scale: 7—strongly agree, 6—agree, 5—slightly agree, 4—neither agree nor disagree, 3—slightly disagree, 2—disagree, 1—strongly disagree. The result obtained indicates a general level of satisfaction with life. Low, average and high levels of life satisfaction were distinguished using sten norms.

### Statistical Analysis

Statistical analysis was performed using the SPSS Statistics 24.0 statistical package (IBM, Armonk, NY, USA). The Kolmogorov–Smirnov test, the Shapiro–Wilk compliance test, and Pearson’s test of independence were used. The statistical significance of the results was assumed at 95% probability (*p* < 0.05). Correlations at 99% probability (*p* < 0.01) and 99.9% probability (*p* < 0.001) were also determined. 

## 3. Results

### 3.1. Sociodemographic Data

The mean age of the respondents was 29.22 ± 3.88 years (range of 20.00–42.00). The vast majority of the women (125; 69.06%) lived in cities with a population of >100,000. The women with bachelor’s degree constituted the largest percentage of the study sample (133; 73.48%). The employed women constituted more than half of the study sample (91; 50.28%). Most women were married or had partners (168; 91.71%) (Table 1).

### 3.2. Pregnancy Data

The most numerous women were those in the third trimester of pregnancy (123; 67.96%), in the first pregnancy (137; 75.69%), and not having a child (137; 79.56%). Among the women for whom it was another pregnancy, 24 (64.86%) women delivered vaginally, and 13 (35.14%) women had cesarean section. More than half of the respondents (22; 59.45%) were women whose husbands/partners participated in the previous delivery/deliveries. The women had children aged from 7 to 288 months (Table 2 and Table 3).

**Figure 1:**
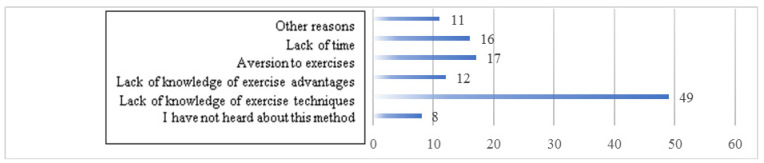
Reasons for not doing pelvic floor muscle exercises.

The most common reason for not doing Kegel’s exercises was the lack of knowledge of how to do exercises (49; 53.26%). Other causes were forgetting, laziness, no need due to a planned cesarean section, and unsuccessful attempts to exercise (Figure 1).

### 3.3. Relationship Data 

The mean age of the surveyed women’s partners was 31.00 ± 4.87 (range of 23–54). The time of living together was 1–288 months (Table 4).

The majority of women (146; 80.66%) were interested in the topic of sexual intercourse during pregnancy, and every fifth woman (35; 19.34%) was not interested in these issues. In most cases, the women sought the knowledge concerning sexual intercourse in pregnancy from professional sources—gynecologist (99; 68.75%), and also from unprofessional sources (105; 71.92%)—the Internet. Only seven (4.86%) women drew their knowledge from midwives, and six (4.17%) from antenatal classes. The largest percentage of the study sample were women (162, 89.50%) who denied that the frequency of sex increased. Nineteen (10.50%) women reported the higher frequency, and 135 (74.59%) reported the lower frequency of sexual activity compared to the state before pregnancy. 

### 3.4. The Level of Sexual Satisfaction of Pregnant Women

The studied women obtained results in the range of 1–10 sten, with 5 sten indicating an average level of satisfaction with closeness and caress, 6 sten—an average level of overall sexual satisfaction, and 7 sten—high satisfaction with sexual intercourse (*p* < 0.001). Raw results were divided into three levels of sexual satisfaction: 1–4—low level, 5–6—average level, 7–10—high level. The most numerous women were those with a high level of overall sexual satisfaction (77; 42.54%) and satisfaction with sex (81; 44.75%), and women with medium levels of satisfaction with closeness (71; 39.23%) and caress (86; 47.51%) (Table 5).

### 3.5. The Level of Life Satisfaction of Pregnant Women

The studied women obtained sten scores ranging from 1 to 10 (8 sten on average), which reflects their high satisfaction with life (*p* < 0.001). Raw results were divided into ranges corresponding with three levels of life satisfaction: 1–4—low level, 5–6—medium level, 7–10—high level). The most numerous women were those with a high level of life satisfaction (134; 74.03%). The analysis of the data showed that life satisfaction was statistically significantly related to overall sexual satisfaction and satisfaction with closeness, caress, and sex. A higher level of life satisfaction was associated with a higher level of overall sexual satisfaction (*p* < 0.001), a higher level of satisfaction with closeness (*p* < 0.01), a higher level of satisfaction with caress (*p* < 0.05), and a higher level of satisfaction with sex (*p* < 0.001). Pregnant women with a high level of sexual satisfaction had higher levels of life satisfaction (Table 6).

There were no statistically significant relationships between the overall sexual satisfaction and the satisfaction with closeness, caress, and sex in the group of women having children (*p* > 0.05) (Table 7).

The analysis of the women’s satisfaction with life in each trimester of pregnancy showed a statistically significant relationship between the life satisfaction and trimester of pregnancy. The level of life satisfaction in pregnant women (*p* < 0.05) decreases as pregnancy progresses (*p* < 0.05) (Table 8).

The analysis of the changes in the frequency of the intercourse compared to the state before pregnancy demonstrated that the largest percentage of the study sample were women who denied that the frequency of sex increased (162; 89.50%). A higher frequency was only reported by 19 (10.50%) women. The surveyed women most often indicated that the reason for the increase in sexual activity was that they felt more attractive. Important factors were also the reduced workload and no pressure associated with trying to have a baby (6; 31.58% in both cases) (Table 9).

Three quarters of the women (135; 74.59%) reported a decrease in the frequency of intercourse compared to the state before pregnancy. The most common reason for reducing the frequency of intercourse during pregnancy was pregnancy complaints (60; 44.44%). The women were also afraid that there could be damage to the baby during intercourse (38; 28.15%). They admitted that they felt less attractive (38; 28.15%), and received no interest from their partners (31; 22.96%) (Table 10). 

A statistically significant relationship was observed between being in the first pregnancy and reduced intercourse compared to the pre-pregnancy period. The decrease in the frequency of intercourse applies mainly to women who are in the first pregnancy (*p* < 0.05) (Table 11). 

No statistically significant relationships were observed between the overall sexual satisfaction and satisfaction with closeness, caress, and sex and the trimester of pregnancy (*p* > 0.05) (Table 12).

A statistically significant relationship was observed between the pelvic floor muscle exercises and sexual satisfaction in the dimension of closeness and caress. The women who performed Kegel’s pelvic floor muscle exercises had a higher level of satisfaction with closeness (*p* < 0.05) and caress (*p* < 0.05). There was no relationship between doing the exercise and overall sexual satisfaction and satisfaction with sex (*p* > 0.05) (Table 13).

No statistically significant relationships were observed between searching for information on intercourse during pregnancy and the overall sexual satisfaction or satisfaction with closeness, caress, and sex (*p* > 0.05) (Table 14). 

## 4. Discussion 

People’s sexual health in every period of life has been arousing great interest among the researchers of many scientific disciplines for years. However, there are few publications discussing the aspect of sexuality during pregnancy. The available studies differ mainly in terms of research methods and instruments used in them, as well as the religious, cultural, social, and ideological aspects. In the study conducted by Aydin et al. [4], using the Female Sexual Function Index (FSFI), as many as 91% of pregnant women met the criteria for sexual dysfunction compared with 68% of non-pregnant women. Such a large percentage of pregnant women with sexual disorders (including lust, arousal, orgasm, or sexual pain) should be an impulse for modern medicine to meet the expectations of women [5]. In our study, pregnant women showing a high overall level of sexual satisfaction (7–10 sten) constituted only 42.54% (77) of all participants, while 40 (22.10%) women had a low level of sexual satisfaction (1–4 sten). Different results were reported by Huras et al., who informed that only 13% of women were fully satisfied, and 25% were dissatisfied with their sexual life in pregnancy [6]. Other authors compared pregnant and non-pregnant women, determining that the levels of satisfaction (91.08% vs. 67.61%, *p* = 0.0001) and desire (51.52% vs. 48.48%, *p* < 0.001) were higher in the second group of women [4,7]. Sexual satisfaction is undoubtedly an important element indicating sexual well-being or the lack of it, as well as the overall quality of life [8]. In their study of 5582 adults of different sexes and ages, Schmiedeberg et al. observed a relationship between the level of sexual satisfaction and the level of life satisfaction [9]. We noticed that this tendency also continues during pregnancy. A higher level of satisfaction with life was associated with a higher level of overall sexual satisfaction (*p* < 0.001), a higher level of satisfaction with closeness (*p* < 0.01), a higher level of satisfaction with caress (*p* < 0.05), and a higher level of satisfaction with sex (*p* < 0.001). Similar results were reported by Ferreira et al., who found that women with a low quality of life also had a low level of sexual satisfaction [10]. Physical and emotional changes occurring in this period may result in a decline in the level of life satisfaction and the frequency of sexual activity. The majority of women in our study reported the decreased frequency of intercourse compared to the state before pregnancy (74.59%), however, it affected more often women who were in their first pregnancy (*p* < 0.05). Parity had no impact on the level of sexual satisfaction in pregnancy (*p* > 0.05). A decrease in sexual activity was also noted by Sossah, who claimed that the most common reason for reducing the frequency of intercourse during pregnancy was the belief that it can lead to miscarriage [11]. The research conducted in Taiwan has shown that more than half of women thought that pregnancy sex can be dangerous and cause fetal damage [12]. In our investigation, concern for the baby came second on the list of reasons for reducing the frequency of sexual intercourse (28.15%), while the reason that came first was pregnancy complaints (44.44%). The women also felt less attractive (38; 28.15%) and received less interest from their partners (31; 22.96). The frequency of intercourse before pregnancy was also studied by Yeniel A. O. and Petri E., Makara-Studzińska M. et al., Navidian A. et al., and Beiranvand S. P. et al. All these authors reported a reduction in the amount of sexual activity compared to pre-pregnancy levels [13,14,15,16]. Both shifting the age limit at which adults decide to have a child, and conscious childlessness ceased to be considered as unusual. However, the problem of infertility among couples and planning offspring can be associated with a sense of pressure from the family or the environment. Many people of reproductive age live in a hurry, strive for self-development, and pursue a professional career. A small percentage of respondents (10.50%) observed an increase in the frequency of intercourse during pregnancy, which was mostly due to mental comfort, understood as the lack of pressure associated with trying to have a baby and less workload. Completely different reasons for undertaking sexual activity in this period are mentioned by Bello F.A. They include maintaining harmony in marriage and accelerating delivery [17]. It is also interesting that pregnant women showing an increase in sexual activity felt more attractive (63.16%). This suggests that the sense of attractiveness at every stage of life has a huge impact on sexual satisfaction and the frequency of intercourse. Kucharska et al. demonstrated that pregnant women and those who used to be pregnant have a high level of sex appeal and have a positive attitude towards intercourse during this period [18]. Pauleta et al. asserts that as many as 41.5% of women felt less attractive during pregnancy [19]. The awareness of physical and emotional changes occurring in this period could have a positive effect on the self-esteem of pregnant women. Isajeva et al. revealed that except for sexual desire, which remains the same, all other parameters are reduced during pregnancy. Available studies prove that sexuality in pregnancy differs not only from that before pregnancy, but also in individual trimesters [20,21]. This is associated with the dynamic changes occurring in the subsequent stages of pregnancy. The emotional states in the first and the third trimesters of pregnancy seem to be particularly important. Gałązka et al. observed that the decrease in anxiety in the second trimester contributes to higher sexual activity [22]. Huras et al. indicated that the libido of pregnant women significantly decreases in the first and the third trimesters of pregnancy compared to the period before pregnancy. This physiological change probably results in a decrease in sexual activity during this period [6]. In our study, there was no statistically significant relationship between the general sexual satisfaction and the trimester of pregnancy (*p* < 0.05). This relationship was noted by Dafna et al., who found that pleasure from sexual activity decreases with increasing pregnancy [7]. The last trimester of pregnancy is special due to the approaching date of delivery and the resulting fear of pain, previous negative experiences related to delivery, and economic uncertainty. Almost every woman feels the fear of giving birth. Our analysis showed that the level of life satisfaction in pregnant women (*p* < 0.05) decreases as pregnancy progresses. The same results were presented by Kang et al., proving that the decrease in life satisfaction in the last trimester has to do with growing antenatal anxiety during this period [23]. However, there are methods to prevent anxiety growing in the third trimester and its effects, namely proper preparation for childbirth (e.g., at antenatal classes), and social support [24]. Gebuza et al. emphasize the importance of support received by a pregnant woman in the last trimester of pregnancy, also showing that it positively affects her assessment of life satisfaction [25]. Taking care of health and physical activity in the periconceptional and perinatal periods are also factors that affect female sexuality. An important part is played by pelvic floor muscles that are responsible for the functioning of the organs within them. Healthy, fit muscles allow a woman to derive satisfaction from intercourse, because well upplied tissue is better innervated, which allows deeper sensations and enhances sexual activity. Unexercised muscles are weakened and slowly degrade. Due to their biological functions, such as pregnancy and childbirth, women are particularly vulnerable to muscle dysfunctions, which substantially worsen the quality of life, as they contribute to limiting sexual intercourse or even giving up sexual contacts, which often causes the weakening of marriage ties. It has been demonstrated that pregnant women who were doing Kegel’s exercises had a higher level of sexual satisfaction in the dimensions of closeness (*p* < 0.05) and caress (*p* < 0.05). Based on her study of 726 women, Kocur D. concluded that women who regularly exercise these muscle parts more often reach orgasm [26]. According to this author, 80% of the respondents knew where the pelvic floor muscles were, 73% knew their functions, but only half of the women surveyed had ever exercised them. In addition, Modarres et al. described a positive effect of pelvic floor muscle strengthening on sexual satisfaction. There were significant differences in the levels of sexual satisfaction between the intervention and control groups (*p* < 0.001) [27]. Citak et al. confirmed the positive effect of Kegel’s exercises on female sexual function (*p* < 0.0001), and a significant increase in self-esteem in the intervention group (*p* < 0.0001) [28]. In our research, over half of the study sample did not do pelvic floor muscle exercises (52.49%). The most common reason was the lack of knowledge of the exercise technique (53.26%). Sex education during pregnancy helps to develop the right approach to changes in sexual life, and significantly increases the satisfaction of pregnant women. Shojaa M. et al. showed that the knowledge of Iranian pregnant women is low, which translates into ‘low sexual desire’, ‘fear of harm’, and ‘sexual myths’. Moreover, 73% of women during pregnancy had low libido. None of the women sought advice or information from a doctor or midwife [29]. Navidian et al. provided evidence that sexual attitudes in the intervention group were significantly better than in the control group (*p* < 0.0001) after group sexual counseling. Counseling has changed the traditional perception of sexual activity during pregnancy [15]. In our research, no statistically significant relationship was observed between sexual satisfaction and seeking information on intercourse during pregnancy (*p* > 0.05) [15]. The respondents most often sought knowledge about intercourse during pregnancy from the Internet (71.92%) and from a gynecologist (68.75%). The quality of the information obtained had not been verified, which may be the reason why this knowledge was not reflected in higher satisfaction with sexual activity. The pregnant women least often used information from midwives (4.86%) and antenatal classes (4.17%), which suggests that in their opinion, a midwife is not a reliable and easily available source of information on sexual problems in this period. This has been confirmed by the research of Liu et al., who noticed that women were much more likely to use unprofessional sources (the Internet, friends, other women having children) than the knowledge of people professionally involved in the care of pregnant women [12]. In our study, the majority of respondents sought information on sexual activity in pregnancy, but every fifth woman was not interested in getting information on these issues (19.34%). Babazadeh et al. and Shojaa M. et al. believe that the problem is women’s shyness which restrains them from starting a discussion about sexual intercourse during pregnancy with a doctor or a midwife [29,30]. Sexual satisfaction is undoubtedly an important element of the overall quality of life. In the light of research, it seems that discussing changes in sexuality during pregnancy with couples expecting a baby, as part of counseling for pregnant women, would be a good practice of obstetricians. 

## 5. Conclusions

1. Women who are in their first pregnancy show the greatest need for information support, because this is a group in which the decrease in the frequency of intercourse was most often observed. 

2. Pregnant women who performed Kegel’s exercises showed a higher level of sexual satisfaction. Most of the women who did not undertake this type of exercise during pregnancy indicated a lack of knowledge of this technique as a reason. It seems reasonable to educate women on the structure, function, and significance of pelvic floor muscles, as well as on the advantages and techniques of strengthening them using Kegel’s method. Training on Kegel’s exercises and the presentation of their benefits should be a permanent element of educating women in the preconception period and during pregnancy.

3. Decreased levels of life and sexual satisfaction may be factors that reduce the psychological comfort of pregnant women, disturbing the course of pregnancy, delivery and puerperium. There is a need for comprehensive research in this field.

4. There is a visible need for comprehensive perinatal care, including professionally conducted sexual counseling on issues related to intercourse during pregnancy, the ways to minimize the negative effects of pregnancy complaints, the acceptance of a changed appearance of a woman, and the partners’ mutual support.

## Figures and Tables

**Table 1:** Sociodemographic data.

Variable	*n*	M ± SD	Min–Max	Me
Age	181	29.22 ± 3.88	20.00–42.00	29.00
**Variable**			***n***	**%**
Place of residence	City with a population of >100,000		125	69.06%
	City with a population of 10,000–100,000		32	17.68%
	Rural areas		19	10.50%
	City with a population of up to 10,000		5	2.76%
	Total		181	100.00%
Education	Third-level/Bachelor’s		133	73.48%
	Secondary		40	22.10%
	Vocational		7	3.87%
	Primary		1	0.55%
	Lower secondary		0	0.00%
	Total		181	100.00%
Employment status	Employed		91	50.28%
	Unemployed		79	43.65%
	Studying		6	3.31%
	Studying/employed		5	2.76%
	Total		181	100.00%

*n*—number of participants; M ± SD—arithmetic mean ± standard deviation; Min–Max—minimum–maximum; % of participants.

**Table 2:** Pregnancy data.

Variable			*n*	%
Trimester of pregnancy	1st trimester		4	2.21%
	2nd trimester		54	29.83%
	3rd trimester		123	67.96%
	Total		181	100.00%
First pregnancy	Yes		137	75.69%
	No		44	24.30%
	Total		181	100.00%
Having children	No	First pregnancy	137	75.69%
		Miscarriage	7	3.87%
	Yes	One child	31	17.13%
		Two children	6	3.31%
	Total		181	100.00%
Type of previous delivery (a multiple choice question)	Childbirth by nature		24	64.86%
	Cesarean section		13	35.14%
	Forceps delivery		1	2.70%
Presence of the partner during the previous delivery	Yes		22	59.45%
	No		15	40.55%
	Total		37	100.00%
Kegel’s exercises	Yes		86	47.51%
	No		95	52.49%
	Total		181	100.00%

*n*―number of participants; % of participants.

**Table 3:** The age of the child (months) in the group of women having children.

Variable	*n*	M ± SD	Min–Max	Me
Age of the child (months)	37	80.07 ± 68.86	7.00–288.00	80.00

*n*—number of participants; M ± SD—arithmetic mean ± standard deviation; Min–Max—minimum–maximum; Me—median.

**Table 4:** The mean age of the husband/partner.

Variable	*n*	M ± SD	Min–Max	Me	W	*p*
Age of the partner	181	31.00 ± 4.87	23–54	31.00	0.931	0.000
The time of living together (months)	181	77.99 ± 47.88	1–288	72.00	0.926	0.000

*n*—number of participants; M ± SD—arithmetic mean ± standard deviation; Min–Max—minimum–maximum; Me—median; W—Shapiro–Wilk’s test result; *p*—testing probability.

**Table 5:** Sexual satisfaction of the pregnant women.

Variable	*n*	Min–Max	M ± SD	Me	Z	*p*
Closeness	181	1.00–10.00	5.41 ± 2.04	6.00	0.17	0.001
Caress	181	1.00–10.00	5.43 ± 2.50	5.00	0.20	0.001
Sex	181	1.00–10.00	6.69 ± 2.69	6.00	0.22	0.001
Overall sexual satisfaction	181	1.00–10.00	5.88 ± 1.90	6.00	0.16	0.001
**Variable**					***n***	**%**
Closeness			Low level		52	28.73%
			Average level		71	39.23%
			High level		58	32.04%
Caress			Low level		57	31.49%
			Average level		86	47.51%
			High level		38	20.99%
Sex			Low level		31	17.13%
			Average level		69	38.12%
			High level		81	44.75%
Overall sexual satisfaction			Low level		40	22.10%
			Average level		64	35.36%
			High level		77	42.54%

*n*—number of participants; % of participants; M ± SD—arithmetic mean ± standard deviation; Min–Max—minimum–maximum; Me—median; the Kolmogorov–Smirnov test; *p*—testing probability.

**Table 6:** The level of life satisfaction and the level of sexual satisfaction.

Variables	*n*	M ± SD	Min–Max			Me	Z	*p*
Satisfaction with life	181	7.61 ± 1.81	1.00–10.00			8.00	2.14	0.001
**Variable**							***n***	**%**
Satisfaction with life			Low level				9	4.97%
			Average level				38	20.99%
			High level				134	74.03%
			Total				181	100.00%
**Variables**			**Satisfaction with Life**			**Total**	**χ^2^**	***p***
			Low	Average	High			
The level of sexual satisfaction in the dimension of closeness	Low	*n*	7	13	32	52	15.47	0.01
		*%*	13.46%	25.00%	61.54%	100.00%		
	Average	*n*	2	17	52	71		
		*%*	2.82%	23.94%	73.24%	100.00%		
	High	*n*	0	8	50	58		
		*%*	0.00%	13.79%	86.21%	100.00%		
The level of sexual satisfaction in the dimension of caress	Low	*n*	7	14	36	57	11.59	0.02
		*%*	12.28%	24.56%	63.16%	100.00%		
	Average	*n*	2	18	66	86		
		*%*	2.33%	20.93%	76.74%	100.00%		
	High	*n*	0	6	32	38		
		*%*	0.00%	15.79%	84.21%	100.00%		
The level of sexual satisfaction in the dimension of sex	Low	*n*	7	7	17	31	25.74	0.001
		*%*	22.58%	22.58%	54.84%	100.00%		
	Average	*n*	1	16	52	69		
		*%*	1.45%	23.19%	75.36%	100.00%		
	High	*n*	1	15	65	81		
		*%*	1.23%	18.52%	80.25%	100.00%		
The overall level of sexual satisfaction	Low	*n*	7	8	25	40	20.55	0.001
		*%*	17.50%	20.00%	62.50%	100.00%		
	Average	*n*	1	18	45	64		
		*%*	1.56%	28.13%	70.31%	100.00%		
	High	*n*	1	12	64	77		
		*%*	1.30%	15.58%	83.12%	100.00%		
Total		*n*	9	38	134	181		
		*%*	4.97%	20.99%	74.03%	100.00%		

*n*—number of participants; % of participants; M ± SD—arithmetic mean ± standard deviation; Min–Max—minimum–maximum; Me—median; the Kolmogorov–Smirnov test; χ^2^—Pearson’s test for independence statistics; *p*—statistical significance.

**Table 7:** Relationship between the level of sexual satisfaction and having children.

Variables			Having Children		Total	χ^2^	*p*
			No	Yes			
The level of sexual satisfaction in the dimension of closeness	Low	*n*	38	14	52	1.91	0.39
		*%*	73.08%	26.92%	100.00%		
	Average	*n*	58	13	71		
		*%*	81.69%	18.31%	100.00%		
	High	*n*	48	10	58		
		*%*	82.76%	17.24%	100.00%		
The level of sexual satisfaction in the dimension of caress	Low	*n*	46	11	57	2.12	0.35
		*%*	80.70%	19.30%	100.00%		
	Average	*n*	65	21	86		
		*%*	75.58%	24.42%	100.00%		
	High	*n*	33	5	38		
		*%*	86.84%	13.16%	100.00%		
The level of sexual satisfaction in the dimension of sex	Low	*n*	26	5	31	0.70	0.71
		*%*	83.87%	16.13%	100.00%		
	Average	*n*	53	16	69		
		*%*	76.81%	23.19%	100.00%		
	High	*n*	65	16	81		
		*%*	80.25%	19.75%	100.00%		
The overall level of sexual satisfaction	Low	*n*	32	8	40	0.13	0.94
		*%*	80.00%	20.00%	100.00%		
	Average	*n*	50	14	64		
		*%*	78.13%	21.88%	100.00%		
	High	*n*	62	15	77		
		*%*	80.52%	19.48%	100.00%		
Total		*n*	144	37	181		
		*%*	79.56%	20.44%	100.00%		

*n*—number of participants; % of participants; χ^2^—Pearson’s test for independence statistics; *p*—statistical significance.

**Table 8:** Relationship between life satisfaction and the trimester of pregnancy.

Variables			Satisfaction with Life			Total	χ^2^	*p*
			Low	Average	High			
Trimester of pregnancy	1st	*n*	1	0	3	4	**8.86**	**0.05**
		*%*	25.00%	0.00%	75.00%	100.00%		
	2nd	*n*	0	15	39	54		
		*%*	0.00%	27.78%	72.22%	100.00%		
	3rd	*n*	8	23	92	123		
		*%*	6.50%	18.70%	74.80%	100.00%		
Total		*n*	9	38	134	181		
		*%*	4.97%	20.99%	74.03%	100.00%		

*n*—number of participants; %—percent of the study sample; χ^2^—Pearson’s test for independence statistics; *p*—statistical significance.

**Table 9:** An increase in the frequency of intercourse compared to the state before pregnancy.

Variables		*n*	%
An increase in the frequency of intercourse compared to the state before pregnancy	Yes	19	10.50%
	No	162	89.50%
	Total	181	100.00%
Reasons for increasing the frequency of intercourse during pregnancy		*n*	%
No pressure associated with trying to have a baby		6	31.58%
No worries about getting pregnant		5	26.32%
Less workload		6	31.58%
Fewer chores at home		3	15.79%
More interest from the partner		3	15.79%
Feeling more attractive		12	63.16%
Other factors		2	10.53%

*n*—number of participants; % of participants.

**Table 10:** Decreased frequency of intercourse compared to the state before pregnancy.

Variables		*n*	%
Decreased frequency of intercourse compared to the state before pregnancy	Yes	135	74.59%
	No	46	25.41%
	Total	181	100.00%
Reasons for reducing the frequency of intercourse during pregnancy		*n*	%
Endangered pregnancy		19	14.07%
Feeling less attractive		38	28.15%
No interest from the partner		31	22.96%
Pregnancy complaints		60	44.44%
Vomiting		8	5.93%
Nausea		13	9.63%
Breast tenderness		12	8.89%
Drowsiness		28	20.74%
Constipation		5	3.70%
Urine incontinence		1	0.74%
Headaches		16	11.85%
Spinal pain		14	10.37%
Edema		3	2.22%
Fear that there could be damage to the baby		38	28.15%
Embarrassment associated with the closeness of a baby		16	11.85%
Other factors		27	20.00%

*n*—number of participants; % of participants.

**Table 11:** Relationship between being pregnant for the first time and reducing the frequency of intercourse compared to the state before pregnancy.

Variables			First Pregnancy		Total	χ^2^	*p*
			Yes	No			
A decrease in the frequency of intercourse compared to the state before pregnancy	Yes	*n*	107	28	135	0.37	0.05
		*%*	79.26%	20.74%	100.00%		
	No	*n*	30	16	46		
		*%*	65.22%	34.78%	100.00%		
Total		*n*	137	44	181		
		*%*	75.69%	24.31%	100.00%		

*n*—number of participants; %—percent of the study sample; χ^2^—Pearson’s test for independence statistics; *p*—statistical significance.

**Table 12:** Relationship between the level of sexual satisfaction and the trimester of pregnancy.

Variables			Trimester of Pregnancy			Total	χ^2^	*p*
			1st	2nd	3rd			
The level of sexual satisfaction in the dimension of closeness	Low	*n*	1	14	37	52	0.52	0.97
		*%*	1.92%	26.92%	71.15%	100.00%		
	Average	*n*	2	22	47	71		
		*%*	2.82%	30.99%	66.20%	100.00%		
	High	*n*	1	18	39	58		
		*%*	1.72%	31.03%	67.24%	100.00%		
The level of sexual satisfaction in the dimension of caress	Low	*n*	2	17	38	57	1.50	0.83
		*%*	3.51%	29.82%	66.67%	100.00%		
	Average	*n*	1	24	61	86		
		*%*	1.16%	27.91%	70.93%	100.00%		
	High	*n*	1	13	24	38		
		*%*	2.63%	34.21%	63.16%	100.00%		
The level of sexual satisfaction in the dimension of sex	Low	*n*	2	8	21	31	3.42	0.49
		*%*	6.45%	25.81%	67.74%	100.00%		
	Average	*n*	1	20	48	69		
		*%*	1.45%	28.99%	69.57%	100.00%		
	High	*n*	1	26	54	81		
		*%*	1.23%	32.10%	66.67%	100.00%		
The overall level of sexual satisfaction	Low	*n*	1	9	30	40	1.52	0.82
		*%*	2.50%	22.50%	75.00%	100.00%		
	Average	*n*	1	21	42	64		
		*%*	1.56%	32.81%	65.63%	100.00%		
	High	*n*	2	24	51	77		
		*%*	2.60%	31.17%	66.23%	100.00%		
Total		*n*	4	54	123	181		
		*%*	2.21%	29.83%	67.96%	100.00%		

*n*—number of participants; %—percent of the study sample; χ^2^—Pearson’s test for independence statistics; *p*—statistical significance.

**Table 13:** Relationship between pelvic floor muscle exercises and the women’s sexual satisfaction.

Variables			Kegel’s Exercises		Total	χ^2^	*p*
			Yes	No			
The level of sexual satisfaction in the dimension of closeness	Low	*n*	19	33	52	6.51	0.04
		*%*	36.54%	63.46%	100.00%		
	Average	*n*	32	39	71		
		*%*	45.07%	54.93%	100.00%		
	High	*n*	35	23	58		
		*%*	60.34%	39.66%	100.00%		
The level of sexual satisfaction in the dimension of caress	Low	*n*	22	35	57	7.07	0.03
		*%*	38.60%	61.40%	100.00%		
	Average	*n*	39	47	86		
		*%*	45.35%	54.65%	100.00%		
	High	*n*	25	13	38		
		*%*	65.79%	34.21%	100.00%		
The level of sexual satisfaction in the dimension of sex	Low	*n*	12	19	31	1.16	0.56
		*%*	38.71%	61.29%	100.00%		
	Average	*n*	34	35	69		
		*%*	49.28%	50.72%	100.00%		
	High	*n*	40	41	81		
		*%*	49.38%	50.62%	100.00%		
The overall level of sexual satisfaction	Low	*n*	15	25	40	2.13	0.34
		*%*	37.50%	62.50%	100.00%		
	Average	*n*	33	31	64		
		*%*	51.56%	48.44%	100.00%		
	High	*n*	38	39	77		
		*%*	49.35%	50.65%	100.00%		
Total		*n*	86	95	181		
		*%*	47.51%	52.49%	100.00%		

*n*—number of participants; %—percent of the study sample; χ^2^—Pearson’s test for independence statistics; *p*—statistical significance.

**Table 14:** Relationship between the level of sexual satisfaction and searching for information on intercourse during pregnancy.

Variables			Searching for Information on Intercourse during Pregnancy		Total	χ^2^	*p*
			Yes	No			
The level of sexual satisfaction in the dimension of closeness	Low	*n*	37	15	52	4.43	0.11
		*%*	71.15%	28.85%	100.00%		
	Average	*n*	61	10	71		
		*%*	85.92%	14.08%	100.00%		
	High	*n*	48	10	58		
		*%*	82.76%	17.24%	100.00%		
The level of sexual satisfaction in the dimension of caress	Low	*n*	44	13	57	0.78	0.68
		*%*	77.19%	22.81%	100.00%		
	Average	*n*	70	16	86		
		*%*	81.40%	18.60%	100.00%		
	High	*n*	32	6	38		
		*%*	84.21%	15.79%	100.00%		
The level of sexual satisfaction in the dimension of sex	Low	*n*	24	7	31	1.03	0.60
		*%*	77.42%	22.58%	100.00%		
	Average	*n*	54	15	69		
		*%*	78.26%	21.74%	100.00%		
	High	*n*	68	13	81		
		*%*	83.95%	16.05%	100.00%		
The overall level of sexual satisfaction	Low	*n*	30	10	40	1.39	0.50
		*%*	75.00%	25.00%	100.00%		
	Average	*n*	54	10	64		
		*%*	84.38%	15.63%	100.00%		
	High	*n*	62	15	77		
		*%*	80.52%	19.48%	100.00%		
Total		*n*	146	35	181		
		*%*	80.66%	19.34%	100.00%		

*n*—number of participants; %—percent of the study sample; χ^2^—Pearson’s test for independence statistics; *p*—statistical significance.

## Supplementary Materials

The following are available online at https://www.mdpi.com/1660-4601/17/16/5894/s1, Questionnaire S1: The Sexual Satisfaction Questionnaire.
