# Supplementary material for: Sexual and Life Satisfaction of Pregnant Women
Source: Int J Environ Res Public Health. 2020 Aug 13;17(16):5894. doi: 10.3390/ijerph17165894 (PMC7459482; doi:10.3390/ijerph17165894)
Supplement: Supplementary file 1 [file ijerph-17-05894-s001.pdf]

The Sexual Satisfaction Questionnaire

by Mieczysław Plopa

The questionnaire consists of 10 statements concerning intimate relationships taking place in a close relationship (marriage, partnership) of two people. Please, rate the level of satisfaction that you get with each of the given activities. Please, answer honestly to each of the statements, choosing from five options **(circle the appropriate letter)**. Please tick "Does not appear" **(cross)** at those activities that you do not experience in your current relationship (marriage or partnership). Please, remember, to choose the answer which, in your opinion, is consistent with the actual state of your relationship.

		DOES NOT OCCUR	NO SATISFACTION	LOW SATISFACTION	ADEQUATE SATISFACTION	HIGH SATISFACTION	MAXIMUM SATISFACTION
1.	Caressing by your partner the intimate parts of your body			B	C	D	E
2.	Caressing the intimate parts of your partner's body with your hand		A	B	C	D	E
3.	Feeling your partner's scent		A	B	C	D	E
4.	Intimate conversations with your partner		A	B	C	D	E
5.	Hugging your partner		A	B	C	D	E
6.	Taking into account the wishes of the partner in sexual intercourse		A	B	C	D	E
7.	Dancing with your partner		A	B	C	D	E
8.	Taking into account sexual intercourse techniques proposed by your partner		A	B	C	D	E
9.	Sexual intercourse with your partner		A	B	C	D	E
10.	Having during sexual intercourse with your partner		A	B	C	D	E
